# Interoception in adolescence: Evidence from multidomain self-report of bodily sensations and cardiac behavioral measures

**DOI:** 10.1371/journal.pone.0330317

**Published:** 2026-03-16

**Authors:** Silvia Canino, Valentina Torchia, Erica Dolce, Irene Ruffo, Teresa Iona, Simona Raimo, Liana Palermo

**Affiliations:** 1 Department of Medical and Surgical Sciences, Magna Graecia University of Catanzaro, Catanzaro, Italy; 2 Department of Health Sciences, Magna Graecia University of Catanzaro, Catanzaro, Italy; Father Muller Charitable Institutions, INDIA

## Abstract

Interoception, the sense of inner bodily signals, plays a key role in emotional regulation, cognition and mental health. While its relevance in adulthood has been extensively explored, less is known about how interoceptive  abilities develop during adolescence, a period characterised by significant physical and psychological changes. This study aimed to investigate different interoceptive dimensions in adolescents and adults to better understand the developmental profile of this sense. Fifty-four adolescents (aged 12–14) and 50 adults (aged 25–34) completed the Heartbeat Counting Task (HCT) to assess their actual ability to detect heartbeats, their confidence in this ability, and the confidence-accuracy correspondence, and a questionnaire on the tendency to focus on different bodily sensations. The study also examined where participants localised bodily sensations during the HCT. No clear group differences emerged in the accuracy during the HCT. Also, both age groups exhibited similar body localisation patterns, primarily focusing on the chest during heartbeat detection. However, adolescents showed significantly lower metacognitive awareness of their ability to perceive cardiac signals, and higher focus on interoceptive sensations, as reflected in their higher confidence ratings in the HCT and elevated scores on a questionnaire assessing the perceived frequency of internal bodily sensations. There were no significant correlations among the interoceptive measures in either group, suggesting that they may capture partially distinct aspects of interoceptive functioning and contributing to the ongoing debate on how interoceptive dimensions should be conceptualized and measured. Overall, these findings suggest that, while basic interoceptive detection may be established by early adolescence, the capacity to accurately reflect on these internal sensations continues to mature into adulthood, at least for what attains the cardiac domain. Also, the mismatch observed between adolescents’ heightened bodily focus and their limited metacognitive insight may partly help explain why adolescence is a period of increased vulnerability to mental health difficulties.

## Introduction

Interoception, the sense of the physiological condition of the inner body, is integral to how we experience and interpret bodily signals [1,2]. This sense encompasses sensations and representations of physiological signals, such as the heartbeat, itchiness and air hunger [1]. Research has highlighted the importance of interoception in emotional regulation, cognition and overall well-being [3–8].

Following the most well-known model in the field [9], on a conscious level, interoception can be operationalized along three main dimensions: (i) interoceptive accuracy (IAcc), which refers to performance on objective tasks like heartbeat detection; (ii) interoceptive sensibility (ISe), which is the self-evaluated tendency to focus on interoceptive signals, measured through questionnaires; and (iii) interoceptive awareness (IAw), which is the metacognitive ability to assess how accurately one perceives internal signals, evaluated through the correspondence between confidence and actual performance [9]. This taxonomy is further supported by evidence suggesting differential contributions of these dimensions on cognition (e.g., [3,10]) and their different relations with mental health difficulties (e.g., [11,12]).

While this taxonomy has served as a useful framework for interoceptive research, more recent studies have highlighted its limitations (e.g., [13,14]) – such as the fact that these dimensions are very broad and encompass a number of loosely connected phenomena – and the inconsistency between the conceptualization of these dimensions and how they are measured [13]. In particular, Desmedt et al. [13] have recently proposed differentiating interoceptive dimensions based on their level of specificity. Thus, the authors suggested a hierarchical framework in which the highest level is interoception, and then suggested dividing interoception into broad factors such as interoceptive attention, interoceptive sensing, interoceptive interpretation, and interoceptive memory. Within these broad categories, the authors further distinguish more specific and homogeneous subfactors – for instance, interoceptive attention bias (corresponging to ISe in previous models), interoceptive attention regulation, and interoceptive distracting under the broad factor “interoceptive attention”, and interoceptive detection (corresponding to IAcc in [9]), interoceptive magnitude, and interoceptive localization under the broad factor “interoceptive sensing”. Finally, measure-related subfactors that directly link constructs to the outcomes of specific tasks were also identified. In this sense, the Heartbeat Counting Task (HCT), a measure that, following the taxonomy by Garfinkel et al. [9], has been widely used to measure IAcc, should be understood as “*a measure of the capacity to estimate heart rate via mental counting*”.

In this context, it is important to note that the validity of the HCT, in which participants have to report the number of counted heartbeats, as a measure of IAcc has been increasingly questioned, as performance may reflect participants’ beliefs or estimates about their heart rate rather than their actual ability to perceive heartbeats [13,15–18]. This observation aligns with recent discussions suggesting that the HCT may tap interoceptive beliefs, rather than representing a pure IAcc measure.

Despite this debate on the dimensions of interoception, there is a growing recognition of interoception’s role in shaping psychological functioning. Still, as highlighted in a seminal review on interoceptive development by Murphy et al. [19], our understanding of how interoception develops across the lifespan remains limited. In particular, research explicitly investigating interoception during typical adolescence is still scarce (for a similar argument, see also [20]). Adolescence, however, is a period of life that can be particularly relevant for interoceptive learning, as it is characterised by significant body changes [19,21]. Adolescence is also marked by significant maturation of neural circuits involved in processing internal bodily signals, influencing how adolescents perceive and respond to internal states such as hunger, fatigue, and emotional arousal [22]. The maturation of interoceptive processes during this period seems to be linked to the development of self-regulation and emotional resilience [23]. Also, disruptions in interoceptive processing during adolescence can contribute to the onset of mental health disorders, such as anxiety and depression [6].

Recent neurophysiological studies have started to explore how interoception manifests in the adolescent brain. For example, Mai et al. [24] showed that heartbeat-evoked potentials (HEPs), a neural marker of interoceptive processing, are associated with an IAcc measure but not with an ISe measure in adolescents, providing objective evidence of the neurocognitive underpinnings of bodily awareness in this age group.

However, not only have very few studies directly investigated interoceptive dimensions in samples of healthy adolescents and adults, but the existing evidence is also mixed (for an overview, see [19,20]). For example, May et al. [25] reported different neural activity but no differences at the behavioural level between 16 adolescents (15–17 yrs), 19 young adults (20–28 yrs), and 19 mature adults (29–55 yrs) in an interoceptive task probing soft touch. Yang et al. [26], instead, designed a study following the taxonomy by Garfinkel et al. [9] and found higher interoceptive accuracy in the cardiac domain in a sample of 50 adolescents (12–16 yrs) as compared to a sample of 50 adults (23–54 yrs), by using the Eye-tracking Interoceptive Accuracy Task, in which participants view two bouncing shapes and are asked to focus on the one moving in synchrony with their heartbeat. This finding contrasts with the idea that there is a disruption of interoception during adolescence [20]. However, considering that physiological ageing is associated with a reduction in interoception [19,20,27] and that this last study included a sample of adults with a broad age range, it is difficult to determine whether adolescents truly performed better on the task, or whether the effect was due to the inclusion of middle-aged adults in the comparison group. In this study, a subsample of participants (35 adolescents and 21 adults) was also given the Multidimensional Assessment of Interoceptive Awareness [28], a measure of ISe following the authors and some taxonomies (but see [13] for criticism in such a classification for this instrument), and, in this case, adolescents showed a lower tendency to actively listen to the body for insight.

Qualitative findings also suggest that adolescents may experience body awareness in highly individualised ways, shaped by both bodily changes and psychological development. For instance, Pérez-Peña et al. [29] found that adolescents and young adults described their interoceptive experiences as fluctuating and context-dependent, often reflecting their struggles in interpreting bodily cues during times of emotional stress or social pressure. Such subjective accounts further emphasize the need to investigate interoceptive development through both quantitative and qualitative lenses.

Thus, to advance our understanding of interoception development, the current study investigated multiple interoceptive dimensions during adolescence, analysing possible differences from the adult pattern of development. To these aims, healthy adolescents, whose ages ranged between 12 and 14 years, and adults, whose ages ranged between 25 and 34 years, performed a protocol that included objective and self-report interoceptive measures. Adults in this age range represent an optimal comparison group, avoiding confounding effects related to ageing processes. Indeed, several studies have suggested a regular decline in several cognitive skills (e.g., speed of processing, working memory, and long-term memory) starting from the 20s (see [30–32]), including interoceptive processing (for an overview see [27]).

When the present study was designed, the field largely relied on the tripartite taxonomy proposed by Garfinkel et al. [9], which distinguishes IAcc, ISe, and IAw, and more recent conceptual proposals had not yet been published. Accordingly, we developed a protocol addressing these specific dimensions, to which we continue to refer for comparability with prior literature, while also considering recent conceptual developments [13], which recommend a clearer specification of the *type of measurement* (e.g., self-report vs. performance-based) and *bodily domain* targeted by each adopted interoceptive measure.

## Materials and methods

### Participants

Fifty-four typically developing adolescents (33 female participants, 21 male participants; mean age = 13.3 years, SD = 0.75, range 12–14 years) and fifty adults (24 female participants, 26 male participants; mean age = 27.7 years, SD = 2.6, range 25–34 years) participated in this study, based on sample sizes used in previous studies (e.g., [26]). Additionally, a sensitivity power analysis was performed in G*Power 3.1.9.7 [33] for a two-sample t test, two-tailed, with α = .05 and desired power = .80. The analysis showed that the study was powered to detect effects as small as d = 0.56. Thus, any medium-to-large differences between adolescents and adults should have been detectable.

All participants were native Italians from an urban context in southern Italy. Adolescents were recruited from state schools in Calabria (Italy), while young adults were recruited by word of mouth.

All recruited participants showed normal reasoning ability according to the Italian norms of the Raven’s Colored Progressive Matrices (RCPM, [34,35]) or of Raven’s Standard Progressive Matrices (RSPM, for participants aged from 12 to 13 years; [36]) and had normal or corrected to normal vision and no history of neurological or psychiatric conditions.

Participants were recruited between 9 April 2022 and 22 December 2023.

### Ethical approval and consent to participate

This study was part of a larger research project approved by the Calabria Region Ethical Committee (Comitato etico Regione Calabria, Catanzaro, Italy) in accordance with the criteria laid down in the 1964 Declaration of Helsinki.

Before taking part in the study, all adult participants provided written informed consent. Adolescents gave oral assent before the investigation, and their parents gave written informed consent.

### Behavioral testing

#### Assessment of the interoceptive accuracy and beliefs.

All participants completed a performance-based measure of cardiac interoception, namely, the Heartbeat Counting Task (HCT) [37]. The HCT has been traditionally interpreted as an IAcc measure [9] but is increasingly recognized as also reflecting interoceptive beliefs [15,17,18].

Participants, placed in a comfortable position, were invited to relax, close their eyes, and focus on bodily sensations, and were told: “When you hear a voice say “*go*” start counting your heartbeats silently; when you hear “*stop*”, stop counting and tell me the exact number of heartbeats you counted”. They were also instructed not to move during the task and not to perform physical manipulations that could facilitate the detection of the pulse (for example, feeling the beat by testing the pulse). This task was repeated six times, using, for the adult group, time intervals of 25, 35 and 45 seconds separated by two standard rest periods of 20 seconds; shorter intervals of 15, 20 and 18 seconds were used for the adolescents [38]. The rest intervals were of the same duration (20 s) for both groups.

To be sure that the instructions given to participants were clear, they were given a short training interval (10 seconds).

Meantime, real heart activity was recorded using a Bluetooth heart rate monitor (Polar Verity sense, Kempele, Finland), a mobile device that allows easy and non-invasive recording. The heartbeat signals of each participant were recorded and, through comparison with their count made by the participant, the accuracy score was calculated. For each trial, an accuracy score was derived (using the formula of Garfinkel et al. [9]): 1 – (|n real beats – n counted beats|)/ ((n real beats + n counted beats)/2). The accuracy scores obtained were calculated as the average of the six trials, producing an average value for each participant [39]. The inclusion of the reported values (n counted beats) within the denominator prevented an overestimation of the accuracy of performance in people who showed high variance, particularly when more heartbeats were reported than recorded [9].

At the end of the task, participants were asked, “*In which part of your body did you feel your heartbeat during the previous task*?”. Then, an image of a body map was presented (adapted from [40]), and participants were asked to indicate the relevant body areas by circling them. The image also includes a box above the head with the label “nowhere”. Nine body districts were identified: head, right ear, left ear, neck, chest, abdomen, right hand and wrist, left hand and wrist and legs; each body district was assigned 1 when the participant indicated that a specific part associated with the perception of the heartbeat. Zero was assigned to those body districts that were not selected by the participants.

#### Assessment of the interoceptive awareness.

IAw was assessed in the cardiac domain by the correlation between the measure of accuracy in performing the HCT and the degree of confidence in one’s ability to estimate the number of heartbeats in the HCT, expressed by the participant at the end of each trial, on a scale from 0 to 10, where 0 indicated “no perception of heartbeat” and 10 indicated “full perception of heartbeat” (for such methodology see [9]).

#### Assessment of the interoceptive sensibility.

ISe was evaluated considering measures targeting both momentary, state-like beliefs (i.e., confidence ratings), and global, trait-like interoceptive beliefs (i.e., ISe questionnaires; see [14]).

Specifically, for what attains the state-like beliefs, at the end of each HCT trial, the participants rated their confidence in their perceived accuracy of response on a scale from 0 to 10, where 0 indicated “no perception of heartbeat” and 10 indicated “full perception of heartbeat”. Thus, this self-report measure only targets the cardiac domain. The task included six trials, and a mean confidence score was computed for each participant by averaging the confidence ratings across the six trials.

Participants also completed an ISe questionnaire. Specifically, adult participants completed the Self-Awareness Questionnaire (SAQ [41]), while adolescents completed the SAQ-C, an adaptation of the SAQ for children and adolescents [42]. The SAQ and the SAQ-C are self-report questionnaires composed of 35 items developed specifically to evaluate the frequency of common body feelings. Thus, the SAQ and the SAQ-C are self-report measures that target multiple bodily domains. Both versions have been validated in Italian. Items are clustered into two factors, one related to visceral feelings (e.g., “I feel my heart beat in my ears”) and the other to somatosensory feelings (e.g., “I feel my palms sweaty”).

Participants were asked to read each item carefully and to evaluate how often they experienced the described sensation; responses were reported on a five-point Likert scale ranging from never to always (0 = never; 1 = sometimes; 2 = often; 3 = very often; 4 = always). The total score is given by the sum of the responses of all items, providing a score range of 0–140. Higher scores indicate higher levels of ISe.

### Statistical analyses

To verify the normality of data distribution for accuracy scores, we used the Shapiro-Wilk test. Given the non-normal distribution observed in experimental variables, such as the score of the HCT, and considering that the SAQ used Likert-style response items, providing ordinal data, non-parametric statistical analyses were performed.

Specifically, comparisons between the two age groups (adolescents: 12–14 years old *vs.* adults: 25–34 years old) on the different interoceptive measures were performed using the Mann-Whitney U test. Effect sizes for Mann–Whitney U tests were reported using the rank-biserial correlation coefficient r_rb_.

A Chi-squared test was applied to analyse which part of the body was most used during the HCT.

Finally, correlation analyses were conducted to explore the relationship between various interoceptive dimensions within the adolescent and young adult groups. Specifically, Spearman’s correlations were performed to examine the associations between the different interoceptive measures (the accuracy during the HCT, the HCT accuracy-confidence correlation, the mean confidence in the HCT, and the SAQ total score) within each age group.

In addition, for non-significant group comparisons on the HCT, equivalence testing was conducted using the Two One-Sided Tests (TOST) procedure [43]. Equivalence bounds were set to ±Δ (Cohen’s d), where Δ was derived from the data reported by Yang et al. [26] for the adolescent–adult comparison. Equivalence was concluded when both one-sided tests were significant (p < .05).

## Results

Descriptive statistics for the different interoceptive measures are reported in Table 1.

**Table 1:** Descriptive statistics for the interoceptive measures in the groups of adolescents and adults.

Adolescent group				
	*Interoceptive Accuracy and Beliefs*	*Interoceptive* *Awareness*	*Interoceptive Sensibility*	
	HCT SCORE	HCT ACCURACY-CONFIDENCE CORRELATION	SAQ	CONFIDENCE- HCT
Mean (SD)	0.36 (0.4)	0.002 (0.5)	44.8 (19)	7.53 (1.25)
Min -Max	−0.95–0.91	−1–0.96	12-94	4.8–10
**Adult group**				
	***Interoceptive Accuracy and Beliefs***	***Interoceptive Awareness***	***Interoceptive Sensibility***	
	HCT SCORE	HCT ACCURACY-CONFIDENCE CORRELATION	SAQ	CONFIDENCE- HCT
Mean (SD)	0.50 (0.4)	0.24 (0.4)	33.3 (14.3)	6.57 (1.6)
Min- Max	−0.54–0.95	−0.86–0.97	10–73	2.50–9.33

HCT, Heartbeat counting task; SAQ, Self-Awareness Questionnaire.

Concerning the accuracy during the HCT, the Mann–Whitney U test showed no statistically significant difference between adolescents and adults in counting their heartbeats (U = 1055, p = .055; r_rb_ = .22), although adults reported numerically higher accuracy in performing the HCT (see Table 1).

Instead, adolescents exhibited a statistically significantly lower metacognitive awareness of their cardiac interoceptive ability compared to adults (U = 981, p = .033; r_rb_ = .23), as evaluated by the correlation between the accuracy in performing the HCT and the degree of confidence in the ability to estimate the number of heartbeats during this task.

Concerning the ISe, the Mann-Whitney U test showed a significant effect of age group on both the SAQ (U = 843, p < .001; r_rb_ = 0.38) and on the average confidence in the HCT (U = 911, p = .004; r_rb_ = 0.33), with adolescents reporting significantly higher scores than adults.

To further examine whether the non-significant difference in HCT between adolescents and adults reflected a true absence of group differences or a lack of statistical power, we conducted an equivalence test (TOST procedure). Equivalence bounds were set at ΔL = −0.47 and ΔU = 0.47 (Cohen’s d), based on the effect size derived from the study of Yang et al. [26] for the group comparisons between adolescents and adults in their cardiac accuracy measure. The observed effect was small (*d* = −0.35, 90% CI [−0.69, −0.03]) and the TOST procedure was not significant (*t*_l_ (102) = −4.21, *p* < .001; *t*_u_ (102) = 0.59, *p* = .28), indicating that the effect could not be considered statistically equivalent to zero. However, the effect observed in our sample was in the opposite direction to that reported by Yang et al. [26], with adults performing slightly better than adolescents.

Correlation analyses showed no significant associations between the different interoceptive dimensions in both age groups (for *adolescents,* see Table 2; for *adults,* see Table 3).

**Table 2:** Spearman correlation coefficients between the interoceptive measures in the adolescent group.

Adolescent group					
		*Interoceptive Accuracy and* *Beliefs*	*Interoceptive Awareness*	*Interoceptive* *Sensibility*	
		HCT SCORE	HCT ACCURACY-CONFIDENCE CORRELATION	SAQ	CONFIDENCE -HCT
Interoceptive Accuracy and Belief	*r*_*rho*_ *p*	–	−0.07 .62	−0.06 .68	0.18 .20
Interoceptive Awareness	*r*_*rho*_ *p*	–	–	0.16 .27	−0.04 .81

HCT, Heartbeat counting task; SAQ, Self-Awareness Questionnaire.

**Table 3:** Spearman correlation coefficients between the interoceptive measures in the adult group.

Adult group					
		*Interoceptive* *Accuracy and* *Beliefs*	*Interoceptive Awareness*	*Interoceptive Sensibility*	
		HCT SCORE	HCT ACCURACY-CONFIDENCE CORRELATION	SAQ	CONFIDENCE-HCT
Interoceptive Accuracy and Belief	*r*_*rho*_ *p*	–	0.17 .24	−0.26 .06	0.28 .05
Interoceptive Awareness	*r*_*rho*_ *p*	–	–	−0.20 .17	−0.09 .55

HCT, Heartbeat counting task; SAQ, Self-Awareness Questionnaire.

Concerning the two ISe measures, we found no significant associations between the SAQ and the confidence rating in the HCT, both in adolescents (ISe-SAQ and ISe-confidence, r_rho_ = .05, p = .517) and in adults (ISe-SAQ and ISe-confidence, r_rho_ = .104, p = .471).

Finally, a chi-squared goodness of fit test was performed separately for adolescents and adults to examine which body parts were used most frequently during the HCT.

For adolescents, the distribution of selected body parts was significantly different from a uniform distribution (χ²(9) = 146, p < .001). The most commonly used body part was the chest (49.35%), followed by the right hand/wrist (14.29%) and the neck (10.39%). A similar pattern emerged for adults, χ²(9) = 88.0, p < .001, with the chest again being the most frequently selected body part (36.25%), followed by the right hand/wrist (17.5%) and left hand/wrist (16.25%).

To compare the distribution of body part selection between the two age groups (adolescents vs. adults), a Chi-squared test of independence was performed. Body-part selection did not differ significantly across age groups (χ²(8) = 8.63, p = .374). This suggests that the body parts used during the HCT were not significantly different between adolescents and adults.

## Discussion

The study examined interoceptive functioning across multiple dimensions in adolescents, with a focus on potential developmental differences when compared to adults.

These dimensions were conceptualized according to the classical taxonomy proposed by Garfinkel et al. [9], which distinguishes interoceptive accuracy, sensibility, and awareness. This framework was adopted because it represented the prevailing model at the time the study was designed and remains the most widely used reference in the field. At the same time, in line with more recent recommendations [13], we sought to promote conceptual clarity by explicitly stating the *type* of measurement and *bodily domain* addressed by each interoceptive measure included in our protocol.

No statistically significant group differences emerged in estimating heart rate via mental counting (HCT score), whereas a clear difference was observed in the cardiac IAw measure, with adults reporting significantly higher metacognitive insight into their cardiac signals. On the other hand, adolescents scored higher on both measures of ISe targeting trait-like and state-like beliefs: the SAQ and the confidence ratings during the HCT. Taken together, this pattern highlights a dissociation between the subjective experience of bodily sensations and the metacognitive ability to evaluate it.

The lack of a statistically significant group difference in the accuracy score for the HCT contrasts with previous findings by Yang et al. [26], who reported a higher level of cardiac accuracy in adolescents, using the Eye-tracking Interoceptive Accuracy Task. Thus, to further explore whether this non-significant difference reflected a true absence of group differences or a lack of statistical power, an equivalence test (TOST procedure) was conducted. The TOST indicated that the effect could not be considered statistically equivalent to zero, suggesting that a small difference between adolescents and adults cannot be ruled out. Importantly, however, the observed effect was in the opposite direction of that reported by Yang et al. [26], with adults performing slightly better than adolescents in our study.

This difference may be due to the broader age range of the adult sample by Yang et al. [26], which included individuals into middle adulthood, where interoceptive accuracy is thought to decline. By limiting our adult cohort to 25–34 years, we minimised this age-related confound.

Methodological differences (e.g., kind of task) may also have contributed to the discrepant results. Indeed, recent evidence suggests a lack of convergence among interoceptive measures—not only across different bodily domains, but also between performance-based tasks designed to assess the same bodily domain (see, for example, [13,44]).

The finding of lower metacognitive insight about cardiac interoception (our IAw measure following Garfinkel et al. [9]) in adolescents is in line with developmental models of metacognition (e.g., [45]) and supports the idea that, also for what attains interoception, at least in the cardiac domain, the metacognitive abilities mature later than more basic ability, such as the one related to detecting bodily signals.

Conversely, the higher self-reported frequency of a vast range of visceral and somatosensorial bodily sensations (one of our ISe measures following Garfinkel et al. [9]) in adolescents may reflect the heightened bodily attention characteristic of adolescence due to pubertal changes [19] or psychosocial factors. This finding is also interesting in light of studies that suggested that an exaggerated interoceptive sensibility can be dysfunctional (see [11]). Indeed, this increased tendency to notice internal bodily signals may be physiological, but can become fertile ground for the onset of mental health disorders (e.g., anxiety and depression).

The lack of significant correlations between interoceptive dimensions in both age groups replicates previous findings (for adolescents see [24]; for adults see [9]), and aligns with the three-dimensional taxonomy proposed by Garfinkel et al. [9], which conceptualizes these dimensions as relatively independent. Indeed, in our data, even within each age group, objective performance did not correlate with subjective sensibility or metacognitive awareness, confirming that metacognitive or subjective insight into internal states does not necessarily align with actual accuracy.

However, our findings revealed no correlation between the two ISe measures, that is, the SAQ scores and confidence ratings during the HCT, within either group. These findings are in line with the idea that these measures probe different ISe aspects [14] and with recent theoretical accounts [13], suggesting that the low convergence across tasks within the same interoceptive dimension may reflect the fact that these dimensions are too broad and encompass heterogeneous phenomena. Indeed, while the SAQ captures a general, habitual focus on bodily signals, confidence ratings may reflect a momentary, context-dependent judgment of interoceptive certainty [14]. In developmental contexts, this is particularly relevant, as adolescents might report increased general interoceptive sensibility due to physical and emotional changes, without this necessarily translating into higher confidence in specific interoceptive tasks.

Regarding body localisation during the HCT, both adolescents and adults most frequently relied on the chest, followed by the wrists and neck. These patterns significantly deviated from a uniform distribution, indicating consistent preferences for certain bodily areas. However, the similarity between age groups in body part selection suggests that both adolescents and adults use comparable perceptual strategies when attending to internal sensations. This may reflect shared physiological or conceptual representations of interoceptive cues, such as the heartbeat, regardless of developmental stage.

Altogether, the findings contribute to a more comprehensive understanding of interoception in adolescence. They indicate that while basic detection of cardiac signals may already be well established, the ability to reflect on or interpret these sensations continues to develop. The observed mismatch between heightened bodily focus and lower metacognitive awareness in adolescents may have implications for emotional processing and vulnerability to psychological distress.

Given the role of interoception in emotion regulation and psychopathology, these results suggest that interventions aimed at adolescents could benefit from fostering the ability to accurately evaluate and understand internal states.

Despite its contributions, this study has some limitations. The cross-sectional design prevents us from inferring developmental trajectories, and our sample size, although adequate, could be increased to improve statistical power. The validity of the HCT has been questioned, as it may reflect participants’ estimation of their heart rate rather than their actual ability to feel the heartbeats [15,17,46]. Also, our IAcc and IAw measures exclusively targeted the cardiac modality.

Future studies should consider longitudinal designs and additional interoceptive measures that consider different organ systems, including the cardiac, gastric, and respiratory systems (for an overview, see [16]), to enhance our understanding of interoceptive development.
